# Metabolic Requirements of *Escherichia coli* in Intracellular Bacterial Communities during Urinary Tract Infection Pathogenesis

**DOI:** 10.1128/mBio.00104-16

**Published:** 2016-04-12

**Authors:** Matt S. Conover, Maria Hadjifrangiskou, Joseph J. Palermo, Michael E. Hibbing, Karen W. Dodson, Scott J. Hultgren

**Affiliations:** Department of Molecular Microbiology and Center for Women’s Infectious Disease Research, Washington University School of Medicine, St. Louis, Missouri, USA

## Abstract

Uropathogenic *Escherichia coli* (UPEC) is the primary etiological agent of over 85% of community-acquired urinary tract infections (UTIs). Mouse models of infection have shown that UPEC can invade bladder epithelial cells in a type 1 pilus-dependent mechanism, avoid a TLR4-mediated exocytic process, and escape into the host cell cytoplasm. The internalized UPEC can clonally replicate into biofilm-like intracellular bacterial communities (IBCs) of thousands of bacteria while avoiding many host clearance mechanisms. Importantly, IBCs have been documented in urine from women and children suffering acute UTI. To understand this protected bacterial niche, we elucidated the transcriptional profile of bacteria within IBCs using microarrays. We delineated the upregulation within the IBC of genes involved in iron acquisition, metabolism, and transport. Interestingly, *lacZ* was highly upregulated, suggesting that bacteria were sensing and/or utilizing a galactoside for metabolism in the IBC. A Δ*lacZ* strain displayed significantly smaller IBCs than the wild-type strain and was attenuated during competitive infection with a wild-type strain. Similarly, a *galK* mutant resulted in smaller IBCs and attenuated infection. Further, analysis of the highly upregulated gene *yeaR* revealed that this gene contributes to oxidative stress resistance and type 1 pilus production. These results suggest that bacteria within the IBC are under oxidative stress and, consistent with previous reports, utilize nonglucose carbon metabolites. Better understanding of the bacterial mechanisms used for IBC development and establishment of infection may give insights into development of novel anti-virulence strategies.

## IMPORTANCE

Urinary tract infections (UTIs) are one of the most common bacterial infections, impacting mostly women. Every year, millions of UTIs occur in the U.S. with most being caused by uropathogenic *E. coli* (UPEC). During a UTI, UPEC invade bladder cells and form an intracellular bacterial community (IBC) that allows for the bacteria to replicate protected from the host immune response. In this study, we investigated genes that are expressed by UPEC within the IBC and determined how they contribute to the formation of this specialized community. Our findings suggest that galactose is important for UPEC growth in the IBC. Additionally, we found that a gene involved in oxidative stress is also important in the regulation of a key factor needed for UPEC invasion of bladder cells. These results may open the door for the development of treatments to diminish UTI frequency and/or severity.

## INTRODUCTION

Uropathogenic *Escherichia coli* (UPEC) accounts for over 85% of reported community-acquired urinary tract infections (UTI) (1). These painful and economically costly infections affect approximately 50% of women at least once during their lifetime (2). In the murine cystitis model, initial colonization is dependent upon the mannose-binding adhesin, FimH, at the tip of type 1 pili (3). FimH binds to mannosylated glycoproteins on the superficial umbrella cells of the urothelium, mediating colonization and triggering subsequent bacterial internalization into the bladder epithelial cells (4, 5). Once inside the epithelial cells, UPEC bacteria are protected from host innate immune defenses, and a single bacterium can replicate to 10^4^ or more bacteria within hours after invasion, forming biofilm-like intracellular bacterial communities (IBCs) (6, 7). Similarly to extracellular biofilms, IBC formation is transient and terminates in a dispersal stage, during which bacteria filament and escape the infected host cells, spreading to neighboring (naive) host cells, where the IBC cycle can be repeated (8). Numerous host defenses against this process, including inflammasome activation and programed urothelial exfoliation and bacterial expulsion via a TRPML3-mediated mechanism, have been uncovered (9–11).

IBCs and bacterial filaments have been documented in urine from women suffering acute UTI 1 to 2 days after sexual intercourse but not in healthy controls or infections caused by Gram-positive organisms, which do not form IBCs (12). In children, the presence of IBCs was predictive of future recurrences (13, 14). Mouse model studies have shown that the ability of UPEC strains to form IBCs allows UPEC to persist in the face of a stringent population bottleneck during acute cystitis, leading to a range of infection outcomes such as the formation of quiescent intracellular reservoirs (QIRs) or the development of chronic cystitis, which is characterized by persistent high-titer bacteriuria (>10^4^ CFU/ml) and high-titer bacterial bladder burdens (>10^4^ CFU) 2 or more weeks after inoculation, accompanied by chronic inflammation (7, 15). During chronic cystitis, luminal bacterial replication is accompanied by persistent lymphoid aggregates in the bladder lamina propria and urothelial hyperplasia with a lack of superficial facet cell terminal differentiation (15). The same histological findings of submucosal lymphoid aggregates and urothelial hyperplasia have been observed in humans suffering persistent bacteriuria (16). Additionally, similarly to what is seen in mice, soluble biomarkers engaged in myeloid cell development and chemotaxis were discovered that are predictive of future UTI recurrence under conditions in which levels are elevated in the sera of young women with UTI (16). These studies demonstrated the ability of the chronic cystitis model to reflect and predict findings related to recurrent UTI (rUTI) risk in women. Collectively, these studies indicated that observations made in murine infection models provide insights into human infection. Therefore, thoroughly understanding the bacterial mechanisms important in mouse models of UTI is highly likely to give important insights for designing antibiologics with antibiotic-sparing therapeutic potential in treating and preventing clinical UTI.

In murine models of infection, approximately 10^7^ bacteria are transurethrally instilled in the bladder (17, 18). Of these, only about 1,000 to 10,000 bacteria become internalized, and only ~1% of internalized bacteria escape into the cytoplasm and initiate the IBC cascade (7). Factors that lead to bottlenecks during the course of infection contribute to reductions in the diversity of the initial bacterial inoculum. These factors include micturition, exfoliation, expulsion, and the innate immune response (9, 10, 16). Such bottlenecks prevent the use of large-scale *in vivo* transposon screens to identify factors involved in mediating IBC expansion and UTI progression and have thus impeded substantial progress in this area of UPEC pathogenesis research. To circumvent this obstacle, previous studies took advantage of *in vitro* screening assays targeting extracellular biofilm formation or assessed the expression of known virulence factors (*hlyA*, *chuA*, *sitA*, *ybtS*, *iron*, *iroB*, *entE*, *entF*, *fepA*, *exbB*, *tonB*, *feoA*, and *ompA*) during IBC development via quantitative PCR (qPCR) (19–21). In addition, several studies have probed the metabolic requirements of UPEC during acute UTI and their results have converged to suggest that UPEC bacteria require aerobic respiration and completion of the tricarboxylic acid (TCA) cycle (despite UPEC strains being facultative anaerobes) and that UPEC primarily rely on amino acid utilization during acute UTI (22–24).

In this study, we utilized a microarray-based approach to perform a genome-wide transcriptional profiling of UPEC 6 h postinoculation (hpi) into the mouse bladder. At that time point, the majority of UPEC are intracellular, with smaller proportions existing extracellularly attached to host cells or in the bladder lumen (6, 8). Our analysis revealed that within 6 hpi, 120 genes were differentially expressed in UPEC compared to the inoculum, which was grown under type 1 pilus-inducing conditions. As expected, and as previously reported, genes involved with siderophore synthesis were dramatically upregulated, indicating that bacteria are starved for iron within the IBC (20, 24). Additionally, several genes associated with alternative sugar metabolism processes, including *lacZ* and *srlA*, were upregulated, suggesting the absence of glucose and the presence of nonglucose sugars for utilization. These data corroborated previous circumstantial observations of *lacZ* upregulation in IBCs, based on strong X-Gal (5-bromo-4-chloro-3-indolyl-β-d-galactopyranoside) staining of IBCs indicative of beta galactosidase activity within the intracellular bacterial community (17, 18, 25). Further characterization of a *lacZ* mutant that exhibited reduced galactose utilization indicated that this mutant was defective in aspects of IBC formation and the subsequent ability to cause chronic cystitis. Analysis of the previously uncharacterized *yeaR* gene, also captured in our arrays, revealed a role for this gene in mediating type 1 pilus production and oxidative stress responses, suggesting a role in adherence and invasion, as well as in responding to the innate immune response. Our analysis of the IBC transcriptome also identified several similar transcriptional profiles found in studies that have probed the UPEC transcriptome of bacteria collected from the urine of patients diagnosed with a UTI; however, those studies did not screen for the presence of IBCs (26–28), which can be sloughed into the urine, depending on the timing of the onset of IBC formation and collection of the urine (12). Combined, our findings further our insights into conditions within the unique intracellular niche of IBCs and provide direction for future studies focused on the development of therapeutics targeting UPEC UTIs (both acute and chronic/recurrent).

## RESULTS

### The transcriptional profile of UPEC during acute UTI.

We investigated the transcriptional profile of UPEC at 6 h postinfection in the C3H/HeN mice. Previous studies have shown that the 6 h time point is the point at which the majority of UPEC bacteria are participating in IBC formation within the bladder epithelial cells (8). For our studies, mice were transurethrally inoculated with the clinical cystitis isolate UTI89 and sacrificed 6 hpi (29). Microscopic analysis validated that IBCs with approximately the same size and shape were present in the superficial umbrella cells of the bladder, and gentamicin protection assays revealed that only 20% of the total CFU recovered were luminal, whereas 80% of the population was intracellular (see Fig. S1 in the supplemental material) (8, 25). RNA was extracted from bladders after extensive washing to remove luminal bacteria and was DNase treated, reverse transcribed (RT), and subjected to microarray analysis using UTI89-specific Affymetrix chips (24). For comparison, RNA was harvested from the bacterial inoculum, which was grown statically at 37°C, as described previously (30). Analyses, after false-detection-rate correction, revealed a total of 120 significantly (*P* < 0.01) differentially regulated genes, or 2.3% of the UTI89 genome. Of these, 40 were upregulated and 80 were downregulated in the bladder. Further analysis, using a 2-fold cutoff value for biological significance, revealed 21 positively and 30 negatively regulated genes (Tables 1 and 2).

**TABLE 1:** Genes positively regulated 6 h postinfection

Locus	Gene name	Fold change	Function^a^
UTI89_C4028	*chuA*	13.6170702	OM hemin receptor
UTI89_C4030		12.62532234	Hypothetical protein
UTI89_C3064	*srlA*	8.533024788	PTS, glucitol/sorbitol-specific IIC2 component
UTI89_C4027	*chuS*	8.363228798	Putative hemin/Hb transport protein
UTI89_C2178	*ybtS*	6.132778168	Salicylate synthase
UTI89_C4033	*chuT*	5.365860462	Putative periplasmic binding protein with FepB/HutB regions
UTI89_C1122	*iroB*	5.215178967	Putative glucosyl transferase
UTI89_C1995	*yeaR*	4.653666496	Hypothetical protein
UTI89_C0599	*ybdB*	4.367933273	Paal_thioesterase—tetrameric acyl-CoA thioesterase with a hot dog fold and one of several proteins responsible for PA, phenylacetic acid PA degradation in bacteria
UTI89_C0587		3.658836126	MbtH-like protein (domain found in antibiotic synthesis proteins)
UTI89_C1994	*yoaG*	3.380447626	Hypothetical protein
UTI89_C5112	*ybcS2*	3.232487202	Bacteriophage lambda lysozyme-like protein
UTI89_C3386	*gspC*	2.910385132	Putative type II secretion protein GspC
UTI89_C1336	*sitD*	2.890235424	Transmembrane subunit, of PBP-dependent ABC transporters involved in siderophore uptake
UTI89_C1162		2.870128155	Hypothetical protein
UTI89_C0371	*lacZ*	2.52590251	Beta-d-galactosidase
UTI89_C5139		2.265412569	Hypothetical protein
UTI89_C4087	*yhjX*	2.237949848	Putative resistance protein (formate/oxalate antiporter)
UTI89_C4654		2.114337921	Hypothetical protein
UTI89_C2656	*hkbM*	2.097069502	Hypothetical protein
UTI89_C4032		2.055215359	Hypothetical protein

a OM, outer membrane; PTS, phosphotransferase system; Hb, hemoglobin; CoA, coenzyme A; PBP, periplasmic binding protein; ABC, ATP-binding cassette.

**TABLE 2:** Genes negatively regulated 6 h postinfection

Locus	Gene name	Fold change	Function
UTI89_C0970	*ycaC*	−2.046942047	Isochorismatase
UTI89_C3263	*ygfK*	−2.056558996	Putative selenate reductase subunit YgfK
UTI89_C2911		−2.075577539	Hypothetical protein
UTI89_C5059		−2.102592326	Hypothetical protein with predicted transporter domains
UTI89_C2208		−2.113388903	Hypothetical protein with predicted beta-lactamase domain
UTI89_C2246		−2.166165631	Putative phosphotriesterase-related protein
UTI89_C3418	*hybA*	−2.218168479	Hydrogenase 2: Fe-S ferredoxin-type component; participates in the periplasmic electron-transferring activity of hydrogenase 2
UTI89_C4757	*yieN*	−2.240676727	Hypothetical protein
UTI89_C4841	*nrdG*	−2.3253116	Anaerobic ribonucleotide reductase-activating protein: activates anaerobic ribonucleoside-triphosphate reductase under anaerobic conditions
UTI89_C4908		−2.330016629	Hypothetical protein with DoxX domain
UTI89_C2574	*yfbS*	−2.437463493	Putative transport protein
UTI89_C3479	*ygiL*	−2.451883269	Putative fimbrial component
UTI89_C3769		−2.463458955	Hypothetical protein
UTI89_C4235	*yidE*	−2.519812232	Putative transporter
UTI89_C1554		−2.529382149	Hypothetical protein
UTI89_C1183	*yceO*	−2.627212273	Hypothetical protein
UTI89_C3868		−2.645149216	Hypothetical protein
UTI89_C2154		−2.759997253	Hypothetical protein
UTI89_P026		−2.890429712	Microcin immunity protein
UTI89_C3966		−2.915524643	Hypothetical protein
UTI89_C4571	*yjaB*	−2.959889227	Hypothetical protein with N-acyltransferase domain
UTI89_C4147	*cysE*	−2.999317444	Serine acetyltransferase: cysteine biosynthesis
UTI89_C0224	*rrfH*	−3.226337436	SS rRNA^a^
UTI89_C3901		−3.240851237	Hypothetical protein
UTI89_C4015	*yhiP*	−3.672725553	Inner membrane transporter member of the POT family of peptide transporters; probable proton-dependent peptide transporter function
UTI89_C3759		−4.033641779	Hypothetical protein
UTI89_C3283		−4.709429327	Hypothetical protein
UTI89_C4738	*fxsA*	−5.055351	Plasmid-mediated exclusion of bacteriophage T7; interacts with the F plasmid-encoded PifA protein; inner membrane protein
UTI89_C4261	*tnaB*	−6.793467107	Tryptophan permease TnaB
UTI89_C0760		−7.142857143	Hypothetical protein

a SS, single stranded.

Several marked differences provided insight into the conditions experienced by UPEC bacteria within the superficial facet cells of the urothelium. For example, of the 21 transcripts whose levels were increased in the IBC relative to the inoculum (Table 1), 8 are associated with iron acquisition systems, suggesting the bacteria are starved for iron *in vivo* (31). Previous laser capture studies have shown that host tissue surrounding the IBC significantly upregulates genes encoding iron-scavenging proteins, demonstrating innate metabolic immunity and bacterial subversion mechanisms occurring at the host-pathogen interface surrounding the IBC (31). Other reports have also demonstrated that iron acquisition is crucial for UPEC pathogenesis (32–34). Additionally, two genes involved in sorbitol and galactose metabolic pathways were upregulated. *srlA* encodes part of the IIC sorbitol-specific transporter subunit that is involved in sorbitol uptake. *lacZ* encodes beta-galactosidase, which cleaves a linkage between galactose and glucose for utilization of these sugars. The *lacZ* gene is part of the *lac* operon that is tightly repressed as long as there is glucose readily available (35, 36). We had previously suspected the lack of glucose within the IBC, given that IBCs are strongly stained by X-Gal, which acts as a beta-galactosidase substrate, and that its cleavage results a blue stain (25). Thus, the increased expression of these carbon intake/utilization systems suggests that glucose availability was limited and that sorbitol and/or a beta-galactoside might have been present at elevated levels in the area surrounding the IBC. Finally, of the 21 genes upregulated in the IBC, 8, including the *yeaR/yoaG* locus, had no annotated function.

A total of 30 genes were identified as being downregulated in IBCs relative to the inoculum; 21 of those genes were hypothetical or of putative function (e.g., putative transporters) or are uncharacterized (Table 1). Other downregulated genes included those involved with the synthesis and transport of the amino acids tryptophan (*tnaB*) and cysteine (*cysE*). This presumably reflects an abundance of these particular amino acids in the intracellular environment, since both of those pathways are regulated by feedback inhibition (37, 38). The type 1 pilus, an essential virulence factor for UPEC in colonizing the mouse urothelium, was not differentially regulated in our IBC microarray even though these fibers are known to be expressed and critical during IBC development. Thus, the level of piliation under the type 1-inducing conditions of the inoculum is likely equivalent to the expression levels in the IBC.

Microarray results were validated by selection of a subset of genes for qPCR analysis (see Fig. S2 in the supplemental material). For qPCR analysis, we randomly chose 5 positively and 5 negatively regulated genes that displayed statistically significant fold changes in the microarray analysis. This analysis confirmed the differential regulation of these targets, albeit the fold change varied from what was observed in the microarray, presumably because qPCR is generally more sensitive to expression differences than arrays. Interestingly, this analysis revealed that *ybdB*, *yeaR*, and *yoaG* were upregulated more than 30-fold in the IBC.

### Analysis of upregulated genes.

Based on the degree of fold positive change, the top 8 upregulated bacterial genes/operons not previously associated with UTI pathogenesis, *yeaR*, *yoaG*, *ybdB*, *gspC*, *srlA*, UTI89_C4030, *lacZ*, and UTI89_C0587, were chosen for further study. *yeaR*, *yeaR-yeaQ* (*yeaR-Q*), *gspC*, *srlA*, UTI89_C4030, *lacZ*, and UTI89_C0587 deletion mutants were constructed in order to assess their effect on fitness in IBC development and UTI pathogenesis. Repeated attempts to delete the highly upregulated *ybdB* gene failed, suggesting either that there is a complicated genomic structure surrounding this gene or that it is essential for growth of UTI89. Initially, given the dominant role of type 1 pili in the murine cystitis model, the effect of each mutation on type 1 piliation was investigated (see Fig. S3 in the supplemental material). Type 1 piliation was measured under inducing conditions using the hemagglutination assay (30). In a hemagglutination assay, normalized bacteria are serially 2-fold diluted and the titer indicates the maximum dilution still capable of agglutinating guinea pig red blood cells. Mannose acts as a competitive inhibitor of type 1 pilus-mediated adhesion; thus, when the hemagglutination assay is performed in the presence of mannose, type 1 pilus-specific agglutination is inhibited. UTI89 showed a hemagglutinin (HA) titer of 2^8^ but a titer of 2^1^ when mannose was present, demonstrating the presence of mannose-sensitive HA (MSHA). We also analyzed these mutants for defects in UTI pathogenesis by measuring their competitive index (CI) against UTI89 28 days postinoculation (dpi) in the bladder and kidneys. Mutations constructed in the hypothetical UTI89_C4030 and UTI89_C0587 genes displayed no alterations in type 1 piliation and no competitive defect (Fig. S2 and data not shown). Deletion of the type II secretion component *gspC* did not influence type 1 pilus expression but did result in a small defect in kidney colonization as previously reported (39). Mutation of *srlA* did not alter type 1 pilus production but resulted in an ~50-fold competitive defect in bladder infection (see Fig. S4). The two most dramatic phenotypes gleaned from this screen involved genes *yeaR* and *lacZ*, which were selected for further study.

### Analysis of *yeaR*.

The *yeaR-yoaG* locus has previously been shown to be upregulated in response to nitrosative stress due to its regulation by the NarL and NsrR nitrogen-sensing systems (40). YeaR has also been shown to be upregulated when *E. coli* is stressed with cadmium or acidified sodium nitrite (41, 42). However, the biological function of these proteins has yet to be elucidated. YeaR contains a domain similar to the S-adenosyl-l-methionine methyltransferase domain found in TehB that has been shown to be involved in tellurite resistance (43). Tellurite exposure results in the generation of oxidative radicals that damage multiple cellular processes (44). However, the homology between these two proteins is less than 25% and may not correlate to functional similarity. YoaG contains no conserved domains but has been annotated as a putative oxidase. The *yeaR-yoaG* operon is hypothesized to contain 2 other genes, UTI89_C1993, a 40-amino-acid protein with no known domains or putative function, and *yeaQ*, which has no known function but is hypothesized to be a transmembrane glycosylase-associated protein. We did not find UTI89_C1993 or *yeaQ* to be upregulated in our studies.

**FIG 1:**
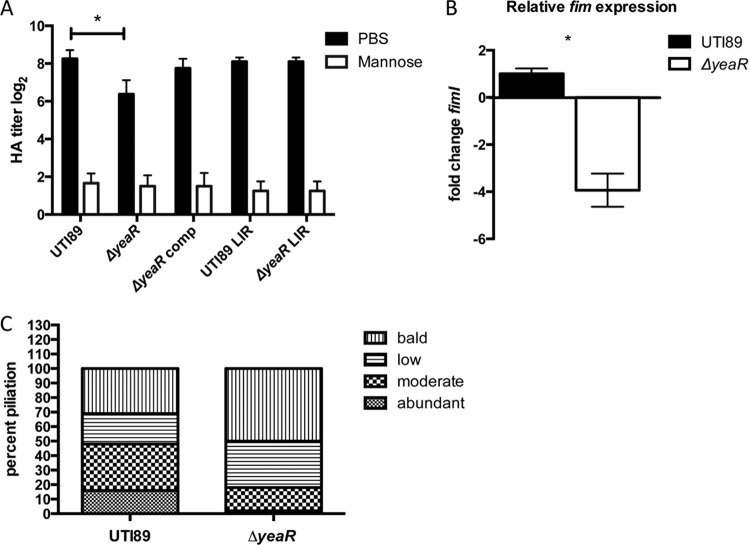
Deletion of *yeaR* decreases type 1 piliation. (A) Type 1 piliation was assessed via hemagglutination for UTI89 and the Δ*yeaR*, Δ*yeaR* comp, LIR UTI89, and LIR Δ*yeaR* mutants. (B) Relative expression levels of *fimI* for the UTI89 and Δ*yeaR* strains were measured by qPCR. (C) Pili were counted on 300 cells per strain. Each cell was assigned one of the general descriptors “abundant,” “moderate,” “low,” and “bald” (no pili) to represent its piliation level. This revealed a shift to a lower piliated population in the Δ*yeaR* mutant that in the UTI89 strain. The cells represented in all panels were grown under conditions of type 1 induction. The representative data shown are from experiments performed in triplicate. Asterisks denote *P* < 0.05 (Student’s *t* test).

To determine if *yeaR* influences the virulence profile of UPEC, a deletion of the *yeaR-yoaG* 4-gene operon was constructed in the UTI89 background, resulting in a Δ*yeaR-Q* strain. This strain had an MSHA titer that is 2-fold to 4-fold lower than that seen with the wild type, suggesting reduced levels of type 1 pili (see Fig. S2 in the supplemental material). Deletion of *yeaR* alone displayed the same 2-to-4-fold-decreased HA titer and was used in subsequent assays to further investigate this single gene (Fig. 1A). The HA phenotype was restored with complementation of *yeaR* on a plasmid. Expression of type 1 pili is governed by the *fimS* promoter, which resides on an invertible element that is oriented by recombinases to give phase ON and phase OFF states (45). We also constructed a *fimS* phase-locked-ON (LIR [left inverted repeat]) UTI89 mutant, which has point mutations incorporated into its left inverted repeat, such that the recombinases that control the inversion of *fimS* can no longer reorient the *fimS* promoter element into the OFF orientation (46). We found that the LIR *fimS* mutation restored the HA titer of the Δ*yeaR* mutant to wild-type levels. We also found that transcript levels of *fim*, as determined by measuring *fimI*, were down 4-fold in the Δ*yeaR* mutant compared to the wild-type strain, consistent with the HA findings (Fig. 1B). Finally, counts of pili on 300 individual cells grown under type 1-inducing conditions were performed using transmission electron microscopy (TEM). This analysis revealed that the Δ*yeaR* strain displayed a shift in population, significantly increasing the number of nonpiliated and/or slightly piliated cells compared to the wild type (Fig. 1C). Together, the results of this analysis suggest that a deletion of *yeaR* causes a decrease in type 1 pilus expression in UTI89.

### *yeaR* influences oxidative stress resistance.

**FIG 2:**
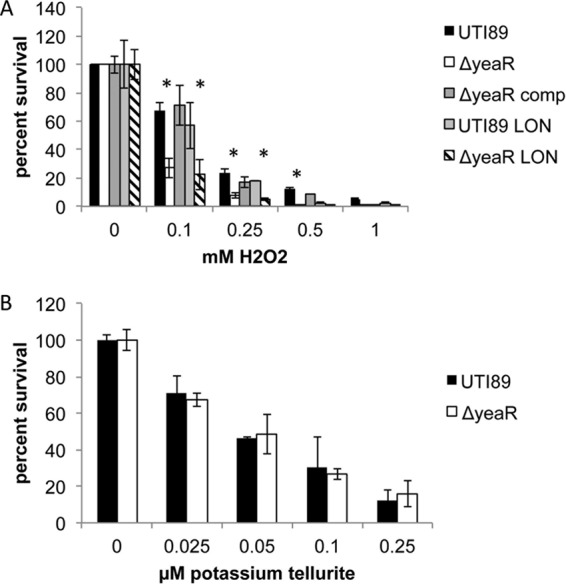
Tellurite and oxidate stress sensitivity. The indicated strains were incubated with increasing concentrations of hydrogen peroxide (A) or potassium tellurite (B) in combination with sodium phosphate buffer for 1 h. Percent survival was calculated against mock-treated cells incubated under the same conditions. The representative data shown are from experiments performed in triplicate. Asterisks denote *P* < 0.05 (Student’s *t* test).

**FIG 3:**
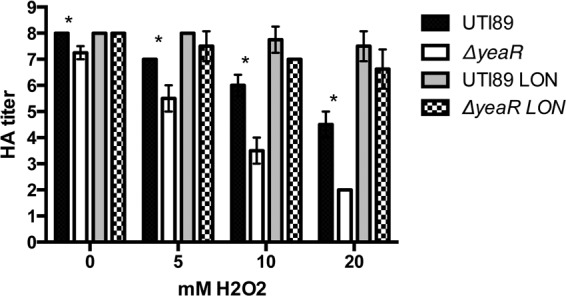
Oxidative stress influences type 1 piliation. The indicated strains were grown under conditions of type 1 pilus induction with the addition of 0, 5, 10, or 20 mM H_2_O_2_ to the growth media. Hemagglutination assays were performed in duplicate. Data represent averages of results of three experiments. Asterisks denote *P* < 0.05 (Student’s *t* test).

In an effort to determine the mechanism by which *yeaR* influences type 1 pilus expression, we first sought to determine if this gene is involved in tellurite resistance as its homology suggests. Incubation with increasing concentrations of potassium tellurite revealed no difference in survival rates between UTI89 and the *yeaR* strain, suggesting that this gene is not required for resistance to that specific heavy metal (Fig. 2A). However, previously published microarrays have suggested that the *yeaR* locus is upregulated during oxidative stress (41). Therefore, to determine if *yeaR* influences UPEC resistance to oxidative stress, killing assays were conducted in 0.1 M sodium phosphate containing 0.1 to 1 mM hydrogen peroxide, revealing that the Δ*yeaR* strain was approximately 3-fold more sensitive to oxidative killing than UTI89 and suggesting a protective role in reactive oxygen sensitivity (Fig. 2B). Hydrogen peroxide-mediated killing is highly dependent on growth conditions. Thus, we investigated the connections between oxidative stress and type 1 pilus production by growing the cells under type 1-inducing conditions, statically in LB, with sublethal concentrations (for LB growth) of hydrogen peroxide (5 to 20 mM). As seen in Fig. 3, increasing concentrations of hydrogen peroxide resulted in an 8-fold decrease of HA titers of UTI89 in 20 mM hydrogen peroxide. Deletion of *yeaR* amplified this effect, resulting in a 64-fold decrease in HA titers in 20 mM hydrogen peroxide. However, LIR UTI89 restored type 1 piliation of all strains. Taken together, these data imply that *yeaR* is involved in resistance to oxidative stress and that oxidative stress results in a reduction of type 1 pilus expression due to orientation of the *fimS* promoter into the OFF position. Therefore, *yeaR* indirectly affects type 1 pilus expression by contributing to the modulation of oxidative stress, which is then sensed by an as-yet-undetermined regulator that modulates the *fimS* switch, thus influencing transcription and production of type 1 pili.

### Decrease in virulence of the Δ*yeaR* strain is due to decreased type 1 piliation.

**FIG 4:**
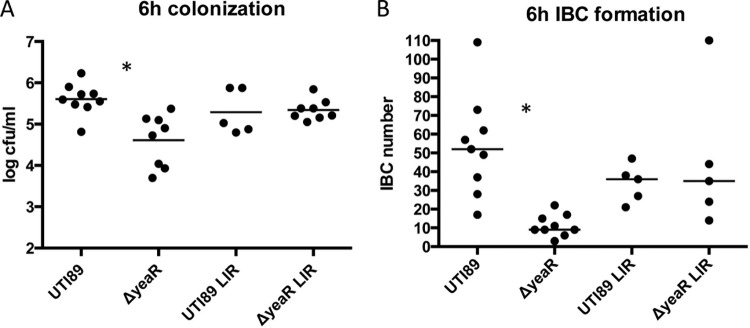
Year influences 6-h IBCs and colonization. C3H/HeN mice were infected for 6 h with 1 × 10^7^ bacteria of the indicated strains. Displayed data are representative of total bladder colonization (A) or IBC enumeration by LacZ staining (B) (*n* = 5 to 9). Asterisks denote *P* < 0.05 (Mann-Whitney *t* test).

**FIG 5:**
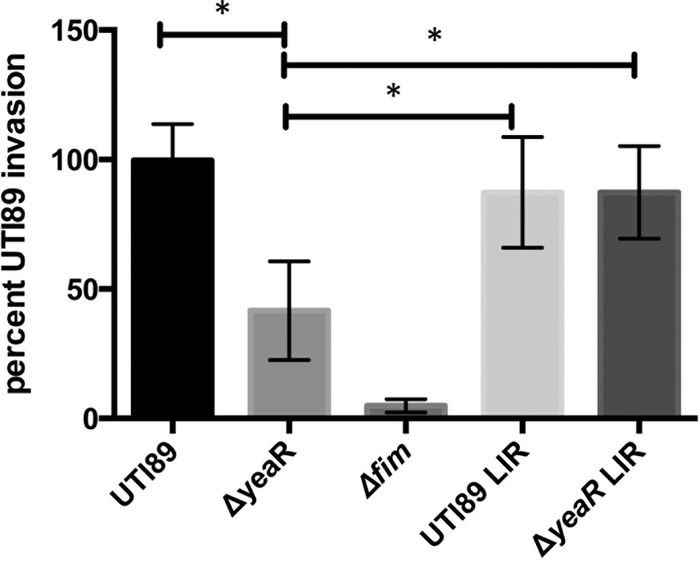
Type 1 pilus-mediated invasion. The UTI89, Δ*yeaR*, Δ*fimA-H* (Δ*fim*), LIR UTI89, or LIR Δ*yeaR* strain was added to the 5637 human bladder epithelial cell line and allowed to invade for 2 h. Subsequent gentamicin treatment and cell lysis allowed the enumeration of internalized bacteria. Asterisks denote *P* < 0.05 (Student’s *t* test).

*yeaR* is highly upregulated in UPEC at the midpoint of IBC formation (6 h). To determine if *yeaR* influenced IBC development and UTI pathogenesis, separately from its effects on type 1 piliation, we inoculated mice transurethrally with each of the UTI89, Δ*yeaR*, LIR Δ*yeaR*, and LIR UTI89 strains and examined IBC formation and CFU at 6 hpi. IBCs were quantitated using a previously described LacZ staining protocol wherein bladders are splayed and stained with an X-Gal solution to visualize punctate blue spots representing IBCs (7). The Δ*yeaR* mutant formed significantly fewer IBCs and CFU per bladder than wild-type UTI89. However, the presence of LIR *fimS* restored these defects, arguing that the primary cause of the decreased fitness of the Δ*yeaR* mutant is decreased type 1 pilus expression (Fig. 4). Our finding that the Δ*yeaR* strain had a 2-fold deficiency in invasion of 5637 bladder cells that was restored to wild-type levels by utilizing the LIR Δ*yeaR* strain or the LIR UTI89 strain was consistent with this conclusion (Fig. 5). As a negative control, a type 1 pilus deletion, *fimA*-*H*, strain was shown to be incapable of invading these cells, confirming that invasion is type 1 pilus dependent. Thus, the decreased levels of type 1 pili seen with the Δ*yeaR* strain result in fewer attachment/invasion events when it is introduced into the bladder, which in turn attenuates IBC formation.

### Defects in galactose metabolism impair IBC development and reduce the incidence of chronic infection.

**FIG 6:**
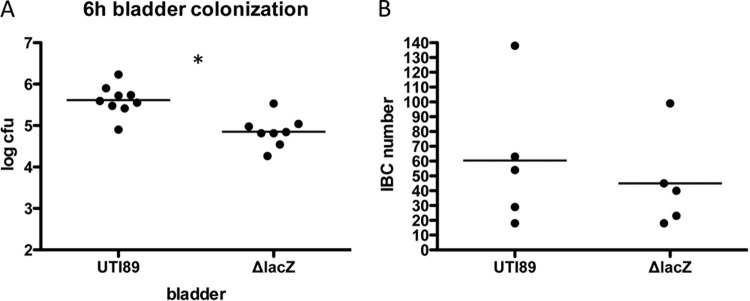
LacZ strain colonization and IBC formation (6 h). C3H/HeN mice were infected for 6 h with 1 × 10^7^ UTI89 or Δ*lacZ* mutant bacteria. Bladders were either homogenized for CFU enumeration (*n* = 8) (A) or stretched and fixed for IBC counting using confocal microscopy (*n* = 5) (B). GFP-expressing strains were used for the IBC counts. The asterisk denotes *P* < 0.05 (Mann-Whitney *t* test).

**FIG 7:**
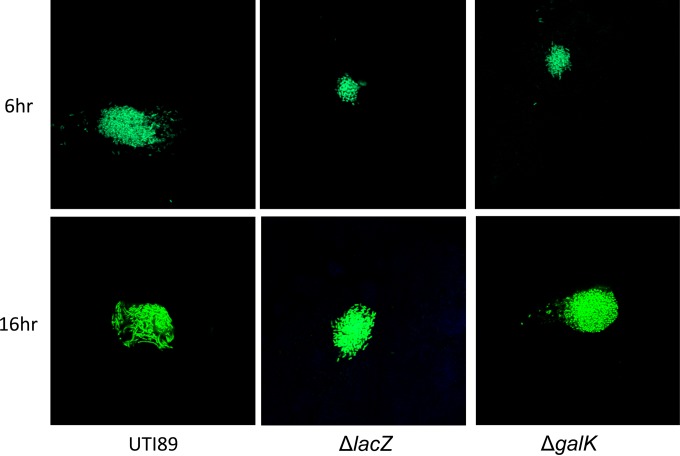
IBC comparisons (6 and 16 h). Representative micrographs depicting GFP-expressing UTI89 and Δ*lacZ* and Δ*galK* mutant IBCs at 6 (top panel) or 16 (bottom panel) h postinfection. UTI89 formed significantly larger IBCs at 6 h than either mutant. At 16 h postinfection, UTI89 begins to filament just prior to dispersal. No filaments were observed at this time point with either the Δ*lacZ* mutant or the Δ*galK* mutant. Images were taken from 5 different mice for each strain.

The *lacZ* gene was also found to be significantly upregulated during IBC development. This is consistent with our finding that X-Gal stains IBCs a punctate blue, arguing that *lacZ* is highly expressed (7, 25). The Δ*lacZ* mutation had no effect on type 1 piliation as determined by hemagglutination assay or invasion of 5637 cells (see Fig. S2 and S5 in the supplemental material). Thus, the effect of the Δ*lacZ* mutation on IBC formation and UPEC virulence was assessed at 6 hpi. In single infection, the growth of the Δ*lacZ* strain was attenuated, resulting in significantly fewer CFU at 6 hpi than seen with UTI89. To investigate the effect of the Δ*lacZ* mutation on IBC formation, strains were transformed with pANT4GFP so that IBC formation could be quantified using confocal microscopy (25). While the Δ*lacZ* strain trended toward producing slightly, but not significantly, fewer IBCs (Fig. 6B), a microscopic and dimensional analysis using Volocity software revealed that the Δ*lacZ* IBCs at 6 hpi were distinctly smaller (average volume of 1,458 µm^3^) than those formed by the wild-type strains (average volume of 4,173 µm^3^) (Table 3). This suggests that beta-galactosidase plays a role in the intracellular growth of bacteria and/or in the maturation of IBCs. Given that LacZ breaks the glycosidic bond of a galactoside for its utilization, we hypothesized that galactose metabolism may be important during IBC development. Thus, this was investigated by generating a Δ*galK* strain, which blocks the conversion of galactose to glucose for metabolic use. In the murine cystitis model, the Δ*galK* mutant displayed a phenotype remarkably similar to that of the Δ*lacZ* strain at 6 hpi: smaller IBCs with dimensions (1,297 µm^3^) analogous to those of the Δ*lacZ* strain (Fig. 7 and Table 3). It has been previously shown that wild-type IBCs reach maturation at 16 hpi and that bacteria within the IBC begin to filament just prior to their dispersal (8, 25). In contrast to UTI89, IBCs formed by Δ*lacZ* and Δ*galK* strains were completely devoid of filaments at that time point (Fig. 7) but were instead continuing to mature in size to between 4,500 and 5,500 µm^3^ (Table 3) with no significant difference between strains. Thus, disrupting galactose metabolism results in slower IBC maturation.

**TABLE 3:** Results of microscopic and dimensional analysis

Time point (h)	UPEC strain	Vol (μm^3^)		SD (μm^3^)	*P* value
		Avg	Median		
6	UTI89	4,173.8	3,735.6	2,538.7	NA
	Δ*lacZ*	1,458.3	551.5	1,735.6	0.008
	Δ*galK*	1,297.6	919.1	1,439.4	0.001
16	UTI89	5,501.9	5,159.3	4,012.3	NA
	Δ*lacZ*	4,541.4	3,099.3	3,809.5	0.599
	Δ*galK*	5,058.4	4,726.7	3,510.2	0.800

a NA, not applicable.

**FIG 8:**
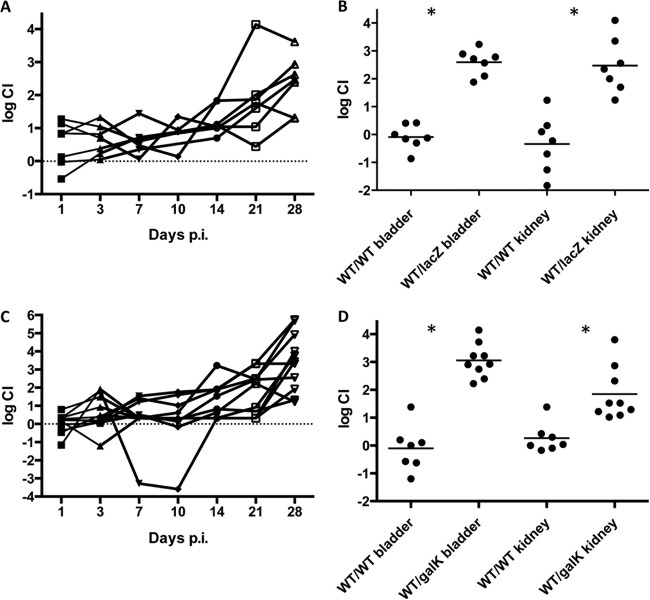
The Δ*lacZ* and Δ*galK* mutations confer a fitness advantage during chronic cystitis. C3H/HeN mice were infected with equal numbers of differentially marked strain UTI89 bacteria in competition with Δ*lacZ* or Δ*galK* mutant bacteria. The log competitive indexes of UTI89 versus the Δ*lacZ* strain (A) and UTI89 versus the Δ*galK* strain (C) were determined in titers of each strain from urine samples collected 1, 3, 7, 10, 14, 21, and 28 days postinfection. Each line represents a single mouse. Log CIs are also displayed for the bladder and kidneys of mice infected with the UTI89 or Δ*lacZ* strain (B) or the UTI89 or Δ*galK* strain (D). UTI89/UTI89 (wt/wt) control competition is also shown (B and D). WT, wild type. Asterisks denote *P* < 0.05 (Mann-Whitney *t* test).

Urine and the UT are traditionally considered to be nutrient-limiting environments with relatively low levels of available sugars/metabolites. As such, we sought to determine if galactose metabolism was important in other stages of cystitis outside the IBC. To address this, we conducted competitive infections using comparisons of the Δ*lacZ* and Δ*galK* strains to wild-type UTI89. When inoculated in numbers equal to those of UTI89, both the Δ*lacZ* mutant and the Δ*galK* mutant were significantly outcompeted during chronic infection (Fig. 8A and B). A significant defect was most clearly demarcated at the later stages of infection, suggesting that galactose metabolism is important during the maintenance of chronic infection. The Δ*lacZ* and Δ*galK* mutants began to be outcompeted by UTI89 after 2 weeks postinfection (average log CI, >1) and after 28 dpi were present at 1,000-fold fewer CFU in the bladder than UTI89. These data demonstrate that galactose metabolism is important for the dynamics of IBC formation and in the maintenance of chronic cystitis.

## DISCUSSION

UPEC association within the host and its ability to establish and to progress to cause UTI involve complex population dynamics between different intracellular and extracellular niches and body habitats, including (i) occupation and maintenance of a reservoir in the gastrointestinal tract (GIT); (ii) colonization of the bladder epithelial surface; (iii) invasion into the urothelial cytoplasm, where rapid replication occurs; and (iv) colonization of the ureters and kidneys (6, 47–50). Thus, UPEC strains must be able to deftly change their expression profile in response to their surrounding microenvironment. Importantly, understanding the UPEC transcriptome during UTI requires consideration of the temporal and spatial niches within the urinary tract that UPEC occupy.

Previous transcriptional analyses of bacteria isolated from the urine of patients with UTI provided valuable insights into the transcriptome of UPEC (27, 28). However, urine likely contains variable UPEC populations, including planktonic bacteria in the urine itself as well as bacteria adhered to or inside sloughed epithelial cells. In addition, the composition of urine varies depending on the nutritional status of the individual and can therefore influence the expression profile of the bacteria growing in the urine. These variables likely explain some of the dramatic variation that has been observed in transcriptional analyses of urine samples. For example, levels of expression of type 1 pili in urine have been shown to differ between different human patients, between individual mice, and between mice and humans (51–53). Such inconsistencies are likely due to differences in the proportions of planktonic bacteria versus bacteria adherent to or present in IBCs within sloughed epithelial cells in the different urine samples. Human urine contains factors that specifically inhibit both expression and function of type 1 pili (54, 55). Planktonic growth in urine induces a phase OFF orientation of the *fim* promoter, thus preventing *fim* expression. Urine also contains inhibitors of FimH function, and the resultant inhibition leads to a further bias in orienting the *fim* promoter toward the phase OFF state (55). However, surface association via type 1 pili favors the phase ON state. While the analysis of the transcriptome of luminal UPEC bacteria has advanced our understanding of UPEC biology, the present study has determined the transcriptional profile of intracellular UPEC, which represents a critical step in the UPEC pathogenic cascade in the bladder.

It is generally hypothesized that factors necessary for the occupation of a niche may be identified by determining those genes which are transcriptionally active in the relevant niche. In humans, collecting bladder biopsy specimens during a UTI is generally contraindicated due to the possibility of increasing the incidence of bacteremia; thus, a thorough analysis of the transcriptional profile of intracellular UPEC requires the application of model systems. In this study, we pursued the transcriptome of the IBC since it one of the unique bacterial niches that UPEC occupies in the urinary tract and is influential in the establishment of cystitis (6). These intracellular communities are transient and undergo several distinct morphological changes throughout their development (8). IBCs are biofilm-like aggregations of bacteria, in comparison to the planktonic bacteria in urine, requiring various surface structures such as type 1 pili, capsule, and OmpA for their formation (6, 21, 56). Therefore, in an attempt to further identify the factors necessary for the establishment of IBCs, we conducted a microarray analysis to elucidate the transcriptional profile of UPEC during mid-IBC growth at 6 hpi and to thereby provide insights into the conditions experienced by the bacteria within the superficial epithelial cells of the bladder. In addition to identifying genes that contribute to IBC development, this analysis also revealed functions that are important for fitness during chronic cystitis, suggesting a role for identified genes in multiple UT niches.

Our examination of the transcriptome of tissue-associated IBCs revealed the upregulation of factors such as galactose metabolism, the *yeaR* locus, and iron acquisition. Interestingly, in a recent study that examined the transcriptional profile of bacteria in urine taken from women experiencing UTI, many of the same systems were also found to be differentially regulated (26). These similarities include the upregulation of iron acquisition systems and of type II secretion as well as, in some cases, the upregulation of *lacZ*, *galK*, and the *yeaR* operon (26, 28). As previously seen for type 1 pilus expression, the expression patterns of *lacZ*, *galK*, and the *yeaR* operon were not universally conserved in the 5 UPEC isolates examined in that recent study (26). However, our work here shows that these factors are important in IBC development and the presence of IBCs was not evaluated in the human urine samples studied. The presence of IBCs in human specimens is variable depending on a multitude of factors, including the progression of the infection and the state of exfoliation of the bladder epithelium, as IBCs form only during the acute stages of infection in the terminally differentiated superficial umbrella cells (12, 13, 26). Results similar to those found in the current study were found in mice in a previous study, which examined the UPEC transcriptome from mouse urine samples mostly collected within the first 24 h of infection, a time period during which numerous sloughed bladder cells containing IBCs are typically present (57).

In experimental infections, UPEC bacteria introduced into the bladder quickly invade the superficial epithelial cells during the first 15 min of infection and begin the IBC cycle (58). Further, at early acute time points (1 to 12 hpi), when micturition and exfoliation result in clearance of the vast majority of inoculated bacteria, the proportions of bacteria remaining are dominated by intracellular and/or tissue-associated bacteria (3), which have been shown to become the founder population of the infection by replication in IBCs (7). Here, and in previous RT-PCR analysis, we have shown that siderophore systems are upregulated in first-round IBCs as well as during growth in urine. Thus, given the early invasion of UPEC into the epithelium and the subsequent rapid growth of the bacteria within IBCs, it is likely that the IBC environment represents a critical trigger for the upregulation of siderophores during UTI (20). This underscores the importance of the IBC cycle in that this niche not only provides protection from the host immune response but also primes the bacteria for extracellular existence in urine.

Our approach to examining the tissue-specific, IBC-enriched transcriptome also revealed alternative metabolic pathways that the UPEC must be utilizing to fulfill its metabolic requirements in the intracellular compartment. One of the key metabolic findings from this study is that glucose is not the primary sugar source within the IBC, as evidenced by the upregulation of *lacZ*. The *lacZ* operon is under tight regulatory control (35). This operon is composed of four genes, *lacI*, *lacZ*, *lacY*, and *lacA* (59). These genes encode the transcriptional repressor, β-galactosidase, lactose permease, and galactoside *O*-acetyltransferase, respectively. Expression of the *lac* operon requires the presence of lactose or a β-galactoside to bind an allosteric site on LacI that alters its conformation, decreasing its affinity for DNA and thus relieving repression of RNA polymerase activity at the *lac* promoter (36). A second condition that must be met for *lac* transcription is that glucose levels must be low in the cell. Low-glucose conditions result in high levels of cyclic AMP (cAMP), which binds catabolite activator protein, allowing it to bind the *lac* promoter and recruit RNA polymerase to transcribe the operon (60). Given that this operon is extremely well characterized and that the input signals are well known, we infer that glucose levels are low within the IBC at 6 hpi and that lactose or a β-galactoside is present. This corroborates previous observations indicating that glycolysis is not essential for UPEC pathogenesis but that gluconeogenesis, TCA cycle completion, and amino acid metabolism are all crucial (23, 24). Additionally, *lacZ* induction is closely associated with galactose utilization, most likely due to LacZ cleaving a galactoside and releasing free galactose, which can then be metabolized by UPEC in an otherwise glucose-limiting environment. The requirement of galactose metabolism for IBC development was confirmed by showing that a *galK* deletion mutant (GalK is an enzyme critical for the conversion of galactose to metabolically usable glucose) was attenuated for IBC formation. Additional evidence for low glucose levels in the IBC environment is that sorbitol intake systems were also upregulated in our analysis. Sorbitol has been reported to be utilized by bladder epithelial cells to regulate the osmotic tension between the epithelium and urine and is crucial for the maintenance of the cell volume in superficial facet cells (61). Therefore, UPEC may be utilizing this highly concentrated sugar alcohol as a metabolite within the IBCs formed in superficial bladder epithelial cells. Deletion of *srlA*, which is involved in sorbitol import, resulted in a significant competitive defect. This suggests that having the ability to process this sugar alcohol provides a fitness advantage to UPEC within the UT. Together, these genetic manipulations and transcriptome profiles have revealed limited-glucose conditions during IBC development and a switch of UPEC metabolism to use galactose and, to some extent, sorbitol, which, in comparison to glucose, requires extra enzymatic processing before it can be metabolized.

Chronic cystitis in mice develops after a week or more of infection and is defined by unchecked luminal bacterial replication and urothelial hyperplasia and the presence of submucosal lymphoid aggregates, a histological pattern similar to that seen in humans suffering chronic UTI (15). The extracellular niche occupied by UPEC during chronic cystitis is thus distinct from the intracellular environment of the IBC that is protected from the immune response. However, we found that both the *lacZ* mutants and the *galK* mutants were dramatically outcompeted by UTI89 during chronic cystitis. It is unlikely that this phenotype can be attributed solely to slower IBC development, since there was no noticeable competitive defect during the first week of competition. This outcompetition during chronic cystitis suggests that the level of sugar metabolites may be limiting during chronic cystitis and that cells able to utilize β-galactosides/galactose are more fit in this environment. Alternately, it is also possible that this pathway is necessary for the assembly of a UPEC virulence determinant. This has been observed previously in *Bacillus subtilis*, where galactose metabolism is essential for the assembly of the exopolysaccharide component of the biofilm matrix, a crucial component of pathogenesis (62). While we cannot discount the possibility that galactose metabolism is influencing biofilm formation or polysaccharide synthesis *in vivo*, mutations in *lacZ* and *galK* did not alter *in vitro* biofilm formation under the standard LB-polyvinyl chloride plate conditions tested (data not shown).

A second theme discovered through our microarray analysis was that UPEC is also responding to oxidative stress as evidenced by *yeaR* being one of the most highly upregulated genes in the IBC. *yeaR* expression has been shown to be induced by heavy metals, which induce oxidative stress, and by reactive nitrogen species (40, 41). Here we show that this gene is involved in resistance to oxidative stress in UPEC and that this oxidative stress influences type 1 pilus expression, thereby attenuating acute pathogenesis. The interconnectedness between oxidative stress and type 1 pilus expression/production has also been documented with IbeA and RpoS (63, 64). Further demonstrating this interconnectedness, bacteria bound to agarose beads in a type 1 pilus-dependent manner displayed a modest upregulation of both the SoxS and OxyR systems in a transcriptional study (65). Additionally, work by Crespo et al. demonstrated that a proper oxidative state of the FimA major pilus subunit is necessary for recognition by the FimC chaperone and subsequent pilus assembly, underscoring the potential effects of oxidative imbalances on pilus biogenesis (66). While higher levels of oxygen tension and oxidative stress are detrimental to type 1 pilus production, the presence of at least some oxygen is required for pilus production. Recent work by Floyd et al. demonstrated that type 1 pili are expressed only in biofilm regions exposed to oxygen (67). Taken together, these data suggest an increasingly evident balance between oxidative stress/sensing mechanisms and type 1 pilus production. Further, UPEC bacteria have evolved mechanisms to overcome this pressure and thus to maintain virulence in hostile environments. Understanding this connection will better elucidate the signals that control type 1 pilus production and UPEC virulence.

Together, these results provide insights into the nutrient and stress conditions faced by UPEC within the IBC. Determining what environmental responses UPEC bacteria mount and what bacterial factors they display during the very early stages of infection will allow the development of therapeutics that could potentially inhibit the establishment of UTI and thereby reduce the occurrence of this highly prevalent infection.

## MATERIALS AND METHODS

### RNA extraction and microarray analysis.

A total of 15 C3H/HeN mice were inoculated transurethrally with 1 × 10^8^ CFU of UTI89 for 6 h. Bladders were aseptically removed and gently washed with phosphate-buffered saline (PBS) to remove extracellular bacteria. Immediately following washing, 3 groups of 5 bladders each were homogenized with 1 ml Tris-EDTA (TE)–100 μg/ml lysozyme and immediately transferred to 1.5 ml of RLT RNA extraction buffer (Qiagen) before mechanical shearing was performed using bead beating and silica beads (two 1-min bead-beating intervals). Homogenates were centrifuged at 10,000 rpm and 4°C for 10 min, and cellular lysates were treated for RNA isolation using columns and reagents from an RNeasy kit (Qiagen). Following extraction, eukaryotic RNA was removed using a MICROBEnrich kit (Ambion) and the remaining “enriched” bacterial RNA was treated with DNase. Exogenous RNA spikes were then added as internal controls for RT and labeling reactions. Resulting cDNA samples were fragmented, biotinylated, and hybridized to GeneChip custom-made genome arrays (Affymetrix UTI89-01a520299F). Data analysis was performed using ArrayStar software. Data were preprocessed using robust microarray analysis (RMA) to eliminate the effects of probe-specific hybridization differences and then normalized using PLYER with a statistical *P* cutoff of <0.01. qPCR was performed as previously described (68) using primers listed in Table S1 in the supplemental material.

### Strains and plasmids.

The cystitis isolate UTI89 was utilized as the wild-type isolate for this study (29). Deletions of *yeaR*, *yeaR-Q*, *gspC*, *srlA*, UTI89_C4030 and UTI89_C0587, *lacZ*, and *galK* were constructed using a λ Red recombinase system and by disruption of the target gene by insertion of a kanamycin cassette (see Table S1 in the supplemental material) (69). All green fluorescent protein (GFP) isolates contained the pANT4 GFP-expressing plasmid which has previously been used in UTI89 (70). Complementation of *yeaR* was accomplished by cloning this gene and its promoter region into the pBAD33 plasmid using BamH1 and HindIII in the orientation opposite that of the arabinose promoter to facilitate native levels of expression (see Table S1).

### Hemagglutination.

Type 1 pilus production was assayed by hemagglutination assay as described previously by Hultgren et al. (53).

### Electron microscopy.

Strains were cultured as described for the mouse infections and were prepared for electron microscopy as described by Rosen et al (71). Briefly, bacteria were absorbed onto Formvar-carbon-coated copper grids for 1 min. Grids were washed in distilled water and stained with 1% aqueous uranyl acetate (Ted Pella Inc.) for 1 min. Images were obtained using a JEOL 1200EX transmission electron microscope (JEOL USA).

### qPCR.

RNA was harvested either from two 24-h (2 × 24) cultures or *in vivo* using an RNeasy kit (Qiagen) as discussed above. Samples were DNase treated before reverse transcription was performed as described previously (68). qPCR was performed with cDNA utilizing a Sybr green system (BioRad) to determine threshold cycle (*C_T_*) values, which were then used to calculate relative fold changes using the ΔΔ*C_T_* method (68).

### Invasion assays.

Bladder epithelial cells of the 5637 cell line (ATCC HTB-9) were seeded into 24-well plates and grown to ~80% confluence, as determined microscopically, in RPMI medium supplemented with 10% fetal bovine serum. Bacteria were then added to the medium at a 10:1 bacterium/host cell ratio for 2 h at 37°C. The medium was then removed and replaced with fresh medium containing 100 µg/ml gentamicin for an additional 2 h. Spent medium was then removed, and the cells were washed 3 times with PBS followed by lysis with 0.1% Triton X-100. Lysates were then plated on LB plates for bacterial enumeration. Experiments were performed in triplicate.

### Mouse infections.

In preparation for inoculation into mice, UPEC strains were grown statically in LB at 37°C overnight and then subcultured at a dilution of 1:1,000 into 10 ml of fresh LB for a second static outgrowth performed at 37°C for 18 to 24 h. From these cultures, 1 × 10^7^ bacteria suspended in PBS were inoculated into lightly anesthetized C3H/HeN mice transurethrally in a 50-µl injection. Infections proceeded from 6 h to 28 days before sacrifice was performed using anesthesia, and the bladders and kidneys were removed and homogenized in sterile PBS. Homogenates were plated for bacterial enumeration. Competitive infections were initiated by mixing 1 × 10^7^ bacteria from each strain into the inoculum. Coinoculated strains carried different antibiotic markers, which allowed the tracking of bacterial titers of the individual strains throughout the time course. Each experiment was repeated in duplicate with separately isolated clones of the noted strains.

### IBC analysis.

At 6 hpi, mice were sacrificed and bladders aseptically removed and splayed for IBC enumeration by *lacZ* staining (7). Alternately, IBC counts for the *lacZ* mutants were determined using GFP-expressing bacteria and confocal microscopy (Zeiss LSM 510). Confocal images were volumetrically analyzed using Volocity software (PerkinElmer).

### Bacterial killing assays.

The indicated UPEC strains were grown under shaking conditions to an optical density at 600 nm (OD_600_) of 1. Bacteria were then diluted to approximately 1 × 10^6^ cells/ml in 0.1 M sodium phosphate. Hydrogen peroxide was added at the designated concentrations followed by incubation for 1 h at 37°C before cells were serially diluted and plated to determine percent survival. Tellurite killing assays were performed similarly with the indicated concentrations of potassium tellurite incubated with bacteria for 1 h at 37°C followed by serial dilution and plating.

### Hydrogen peroxide hemagglutination assays.

Cells were grown as described for mouse infection except for the addition of the indicated concentrations of hydrogen peroxide into the culture at time of inoculation. After 24 h of growth at 37°C, bacteria were subcultured with fresh hydrogen peroxide for an additional 24 h. Bacterial numbers were standardized via optical density and colony enumeration to ensure that similar viable-cell numbers were used for the hemagglutination assay described above.

## SUPPLEMENTAL MATERIAL

Figure S1  Analysis of 6 h UPEC niche occupation. C3H/HeN (*n* = 4) mice were infected for 6 h with 10^8^ CFU of UTI89. Gentamicin protection assays were performed to assess the intracellular and extracellular bacterial niche occupation percentages. *P* = 0.06 (Student’s *t* test). Download Figure S1, TIF file, 0.8 MB

Figure S2  qPCR microarray analysis of differentially regulated genes. To confirm microarray expression patterns, RNA was harvested from the bladders of C3H/HeN mice infected for 6 h with UTI89. cDNA was constructed, and primers specific for the listed genes were used to perform qPCR. Expression changes were determined by comparing the IBC-enriched samples to the inoculum as a control. Fold change was calculated via the threshold cycle (ΔΔ*C_T_*) method. Download Figure S2, TIF file, 1.4 MB

Figure S3  Effect of IBC positively regulated genes on type 1 pilus production. Deletions were constructed in the indicated genes, and the mutants subsequently grown 2 × 24 under type 1 pilus inducing conditions. Hemagglutination assays revealed no differences in type 1 pilus-mediated binding except in the Δ*yeaR-Q* mutant. The asterisk denotes *P* < 0.05 (Student’s *t* test). Download Figure S3, TIF file, 0.7 MB

Figure S4  Sorbitol metabolism provides a competitive advantage during chronic cystitis. Differentially marked UTI89 and Δ*srlA* bacteria were inoculated intravesically into C3H/HeN mice in equal numbers (*n* = 5). Mice were sacrificed 28 days postinfection, and colonization of the bladder and kidneys was assessed, revealing a 50-fold outcompetition of the Δ*srlA* mutant by UTI89. Asterisks denote *P* < 0.05 (Mann-Whitney *t* test). Download Figure S4, TIF file, 0.4 MB

Figure S5  Invasion and *lacZ*. UTI89 or Δ*lacZ* bacteria were added to the 5637 human bladder epithelial cell line and allowed to invade for 2 h. Invasion was quantified following gentamicin treatment and cell lysis. Download Figure S5, TIF file, 0.3 MB

Table S1  Primers used in this study. Table S1, JPG file, 2.7 MB
