# Supplementary figures and images for: Metabolic Requirements of Escherichia coli in Intracellular Bacterial Communities during Urinary Tract Infection Pathogenesis
Source: mBio. 2016 Apr 12;7(2):e00104-16. doi: 10.1128/mBio.00104-16 (PMC4959519; doi:10.1128/mBio.00104-16)

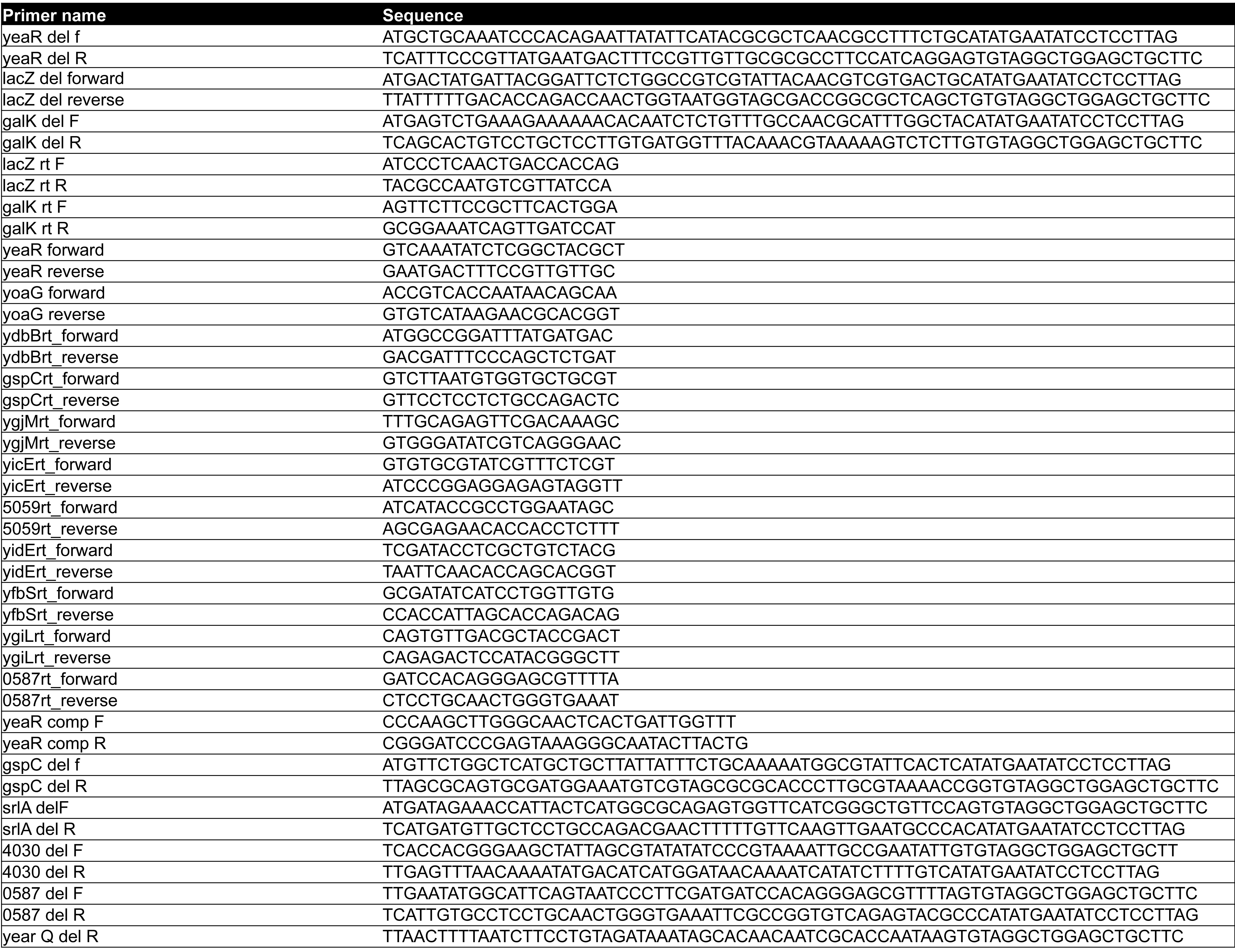

Supplement: Table S1 — Primers used in this study. [file mbo002162771st1.jpg]
